# Effects of animal manure and nitrification inhibitor on N_2_O emissions and soil carbon stocks of a maize cropping system in Northeast China

**DOI:** 10.1038/s41598-022-19592-9

**Published:** 2022-09-08

**Authors:** Dan Dong, Weichao Yang, Hao Sun, Shuang Kong, Hui Xu

**Affiliations:** 1 Key Laboratory of Pollution Ecology and Environmental Engineering, Institute of Applied Ecology, Chinese Academy of Sciences, Shenyang, 110016 China; 2 Jiangsu Collaborative Innovation Center of Regional Modern Agriculture and Environmental Protection/Jiangsu Key Laboratory for Eco-Agriculture Biotechnology Around Hongze Lake, Huaiyin Normal University, Huai’an, 223300 China; 3 University of Chinese Academy of Sciences, Beijing, 100049 China

**Keywords:** Environmental impact, Climate change, Climate-change ecology

## Abstract

The incorporation of animal manure (AM) in soil plays an essential role in soil carbon sequestration but might induce higher soil nitrous oxide (N_2_O) emissions. The use of nitrification inhibitors (NI) is an effective strategy to abate N_2_O emission in agro-ecosystems. However, very few studies have evaluated the effectiveness of applying NI under the combined application of organic and inorganic fertilizers for increasing soil carbon sequestration and reducing N_2_O emissions simultaneously in Northeast China. Here, a four-year field experiment was conducted with three treatments [inorganic fertilizer (NPK), inorganic fertilizer + manure (NPKM), and inorganic fertilizer with NI + manure (NPKI + M)], in a rainfed maize cropping system in Northeast China. Plots of different treatments were kept in the same locations for 4 years. Gas samples were collected using the static closed chamber technique, and nitrous oxide (N_2_O) concentration in gas samples was quantified using a gas chromatograph. Soil organic carbon sequestration rate (SOCSR) was calculated based on the changes in SOC from April 2012 to October 2015. Averaged over the four years, AM incorporation significantly increased soil N_2_O emissions by 25.8% (*p* < 0.05), compared to NPK treatment. DMPP (3,4-dimethylpyrazole phosphate) significantly decreased N_2_O emissions by 32.5% (*p* < 0.05) relative to NPKM treatment. SOC content was significantly elevated by 24.1% in the NPKI + M treatment than the NPK treatment after four years of manure application (*p* < 0.05). The annual topsoil SOCSR for the NPKM and NPKI + M treatments was 0.57 Mg ha^−1^ yr^−1^ and 1.02 Mg ha^−1^ yr^−1^, respectively, which were significantly higher than that of NPK treatment (− 0.61 Mg ha^−1^ yr^−1^, *p* < 0.05). AM addition significantly increased the aboveground biomass and crop yields of maize in the fourth year. Overall, combined application of DMPP, inorganic fertilizer and AM is strongly recommended in this rainfed maize cropping system, which can increase maize yield and SOC sequestration rate, and mitigate N_2_O emission.

## Introduction

In China, agricultural production generates 2.4 × 10^9^ tons of animal manure (AM) each year^1^. The application of AM to soil can help to slow climate change by increasing soil carbon sequestration^2^, improve soil fertility, and tackle environmental problems associated with nitrogen-rich waste management^3^. Nevertheless, AM amendment might cause substantial nitrous oxide (N_2_O) emissions from soils. Intensively fertilized upland soil is one of the anthropogenic sources of N_2_O, and the GWP (Global Warming Potential) of N_2_O is 298 times than that of CO_2_ over a century time horizon^4^. Application of AM will alter soil aerobic conditions, pH, and porosity, and then affect N_2_O emission^5,6^. It is typically believed that, in comparison to inorganic fertilizers, AM provides more labile organic carbon sources for soil microbes, thereby stimulating N_2_O emission from nitrification and denitrification. A global meta-analysis found that the increases in N_2_O emissions caused by manure application might offset the benefit of increasing soil organic carbon (SOC) stocks^7^. In order to mitigate the emission of N_2_O, sustainable agricultural practices must be explored and carried out.

Nitrification inhibitors (NI) have been suggested as a potential option to mitigateagricultural soil N_2_O emissions by the Intergovernmental Panel on Climate Change ^8^. As a recommended NI, 3,4-dimethylpyrazole phosphate (DMPP) has been proved effective at reducing N_2_O emissions from croplands^9^, although the reported abatement of N_2_O emissions ranged from 22 to 77% in maize cropping systems^10,11^. Furthermore, different AM types and managements can make a big difference in the size of subsequent N_2_O emissions^6,12^. In addition, N_2_O emission is also affected by soil characteristics, climatic conditions, and crop management measures^13^. Although several studies have measured the effects of AM-based soil amendments on N_2_O emissions from maize cropping systems in Northeast China—31% of the national maize is grown in the region^14^, most of these studies quantified N_2_O emissions less than one year, which can’t fully capture the inter-annual characteristics of N_2_O emissions^15^. Due to lack of long-term measurement under AM applications, there is still great uncertainty about the quantification and mitigation of N_2_O emissions in the maize cropping system.

To address these gaps, this study presented a long-term observation of N_2_O emission and soil carbon sequestration in a maize cropping system in Northeast China, The main objectives of this study were: (1) to evaluate the combined application of inorganic fertilizer and AM on N_2_O emissions and soil organic carbon sequestration; (2) to test if DMPP can effectively reduce N_2_O emission and increase soil organic carbon sequestration under the combined application of inorganic fertilizer and AM.

## Materials and methods

### Study area and soil properties

A field experiment was established in May 2012 at Shenyang Agro-Ecological Station (41°31′N, 123°22′E) of the Institute of Applied Ecology, Chinese Academy of Sciences, Northeast China. This region has a warm-temperate continental monsoon climate. The mean annual air temperature and annual precipitation are 7.5 °C and 680 mm, respectively. The soil is classified as Luvisol (FAO classification). The soil properties of the topsoil layer (0–20 cm) at the start of the experiment are as follows: SOC = 9.0 g kg^−1^, available NH_4_^+^–N = 1.18 mg kg^−1^; available NO_3_^−^–N = 9.04 mg kg^−1^; Olsen-P = 38.50 mg kg^−1^, available K = 97.90 mg kg^−1^, bulk density = 1.25 g cm^−3^, and pH = 5.8. The determination method of soil was shown in “Soil analysis” section.

### Field experiment

Three treatments were established in this experiment: (1) mineral fertilizers (NPK); (2) pig manure incorporation at a local conventional AM application rate of 15 Mg ha^−1^ yr^−1^ (NPKM, 126 kg N ha^−1^ on dry weight); and (3) NPKM plus DMPP (3,4-Dimethylpyrazole phosphate) incorporation at a rate of 0.5% of applied urea (2.39 kg ha^−1^, 220 kg N/the N content of urea (0.46) × 0.5%) (NPKI + M). The treatments were applied following a randomized design across three replicate field plots (4 m × 5 m). Plots of different treatments remained unchanged in the same locations for 4 years. Each year, the composted pig manure (213 g C kg^−1^ and 22 g N kg^−1^ based on dry weight on average, characteristics of pig manure was listed in Table S1) was broadcasted evenly onto the plots a few days before maize planting, and ploughed to a depth of 20 cm by machine (TG4, Huaxing, China). For the respective treatments, urea (220 kg N ha^−1^ yr^−1^), calcium superphosphate (110 kg P_2_O_5_ ha^−1^ yr^−1^), and potassium chloride (110 kg K_2_O ha^−1^ yr^−1^) were applied on the same day as maize (*Zea mays L*.) was planted. The urea and inhibitor were fully mixed before application.

Maize (cultivar was Fuyou #9) was planted on 3rd May 2012, 3rd May 2013, 6th May 2014, and 10th May 2015, at a spacing of 37 cm and 60 cm between rows. No irrigation was applied throughout the experimental period. Maize was harvested on 13th September 2012, 29th September 2013, 29th September 2014, and 29th September 2015, respectively. At harvest, maize yield and aboveground biomass yield were measured by harvesting all plants (20 m^2^) in each plot. The straw and grain were removed after each harvest and the soil with about 5 cm maize stem was ploughed to a depth of approximately 20 cm in April each year.

Each cropping cycle, therefore, consisted of periods of maize (from May to September) and fallow (from October to April) of the following year.

**Figure 1:**
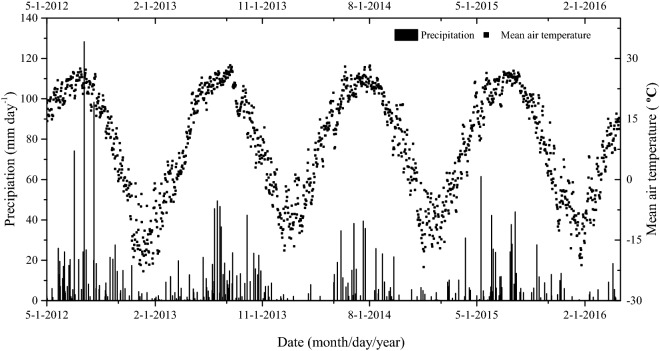
Precipitation and daily mean air temperature during four annual cycles from May 2012 to April 2016 in the experimental field.

**Figure 2:**
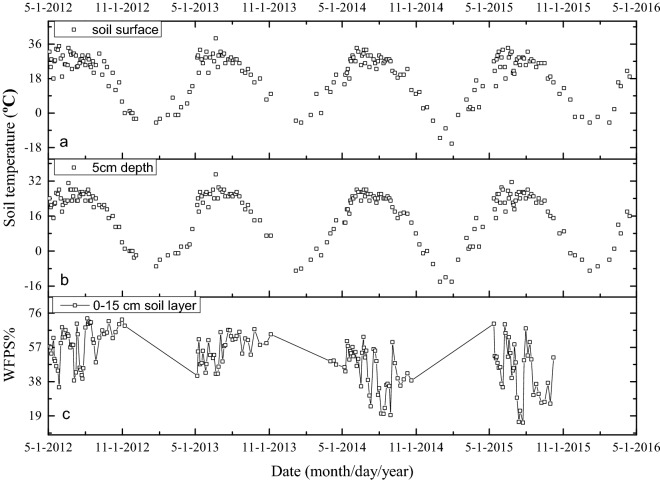
Seasonal variations in soil temperature (at soil surface and 5 cm soil depth) and WFPS% at 0–15 cm depth from May 2012 to April 2016.

The precipitation and air temperature data were acquired from the meteorological station of the Shenyang Agro-Ecological Station. The precipitation during the 2012/2013, 2013/2014, 2014/2015, and 2015/2016 periods were 911.9 mm, 621.7 mm, 485.7 mm, and 585.3 mm, respectively (Fig. 1). 72.3%, 75.5%, 66.5%, and 73.0% of these annual precipitations occurred during maize-growing period, respectively. The mean annual air temperatures in these years were 7.7 °C (− 21.2 to 27.5 °C), 8.1 °C (− 22.7 to 28.3 °C), 9.5 °C (− 21.7 to 28.2 °C) and 9.3 °C (− 17.1 to 27.0 °C), respectively. The soil temperature at a depth of 5 cm varied between − 14 and 35 °C during the four-year period (Fig. 2b). The change trend of soil surface temperature was the same as that of soil temperature at 5 cm depth (Fig. 2a). The mean soil WFPS (0–15 cm) varied between 15 and 73% (Fig. 2c).

### Gas sampling and analysis

The gas was sampled between 3rd May 2012 and 14th April 2016 using a static closed chamber system as described by Dong et al.^16^. Briefly, a stainless-steel chamber base (56 cm length × 28 cm width) was inserted into the soil of each plot to a depth of approximately 10 cm, with its long edge perpendicular to the rows of maize. The top chamber (56 cm length × 28 cm width × 20 cm height) was also made of stainless steel. Gas samples were obtained using a syringe 0, 20, and 40 min after the chambers had been closed between 9:00 am and 11:00 am on each sampling day. Gas samples were collected every 2‒6 days and every 7‒15 days during the growing seasons and non-growing seasons, respectively. The first gas sampling time was on day 1, day 3, day 1, and day 3 after maize planting each year. The N_2_O concentrations in gas samples were quantified using a gas chromatograph (Agilent 7890A, Shanghai, China) with an electron capture detector.

### Soil analysis

The soil temperature and volumetric water content (SVWC) were measured at depth of 0–15 cm using a bent stem thermometer and a time-domain reflectometry (Zhongtian Devices Co. Ltd, China), respectively. SVWC was converted to soil water-filled pore space (WFPS) using the following equation:

1 $${\text{WFPS}} = {\text{SVWC}}/(1{-}{\text{BD}}/{\text{particle}}\,{\text{density}}),$$

where BD is soil bulk density (g cm^−3^). Particle density was assumed to be 2.65 g cm^−3^.

Soil samples from the 0–20 cm layer were collected in each plot in April 2012 (before sowing) and October 2015 (maize harvest) using a 5 cm diameter stainless steel soil sampler. The five soil samples collected from different locations in each plot were mixed thoroughly. Visible roots were removed by hand and the samples were air-dried and sieved using a 0.15 mm sieve. SOC was then quantified using an elemental analyzer (Vario EL III, Elementar, Germany). Soil available NH_4_^+^–N and NO_3_^−^–N were extracted with 2 M KCl and measured colorimetrically using a continuous flow injection analyzer (Futura, Alliance, France)^17^. Soil Olsen-P was extracted with NaHCO_3_ and colorimetrically measured using a spectrophotometer (Lambda 2, PerkinElmer, USA). Soil available K was extracted by 1 M CH_3_COONH_4_ and analyzed with a flame photometer (FP640, Jingmi, China). Soil pH was determined with deionized water (1:2.5) and analyzed using a pH meter (PHS-3C, LeiCi, China) with a glass electrode.

### DNA extraction and real-time quantitative PCR

The soil samples for measuring the abundance of nitrification and denitrification functional genes were collected on May 20, 2015. Soil DNA was extracted with the soil DNA extracted kits (EZNA soil DNA Kit; Omega Bio-Tek Inc., U.S.A.). The copy numbers of nitrification and denitrification functional genes were determined by q-PCR with the Roche LightCyler® 96 (Roche, Switzerland). Additional details about the primers and amplification procedure can be found in Dong et al.^16^.

### Data analysis

The N_2_O flux (μg N_2_O–N m^−2^ h^−1^) is calculated based on the increase of N_2_O concentration per unit chamber area for a specific time interval^18^ as follows:

2 $${\text{F}} = 273/\left( {273 + {\text{T}}} \right) \times {\text{M}}/22.4 \times {\text{H}} \times {\text{dc}}/{\text{dt}} \times 1000$$

where F (μg N_2_O–N m^−2^ h^−1^) is the N_2_O flux, T (◦C) is the air temperature in the chamber, M (g N_2_O–N mol^−1^) is the molecular weight of N_2_O–N, 22.4 (L mol^−1^) is the molecular volume of the gas at 101.325 kPa and 273 K, H (m) is the chamber height, dc/dt (ppb h^−1^) is the rate of change in the N_2_O concentration in the chamber.

Cumulative N_2_O emissions were calculated as follows:

3 $${\text{Cumulative}}\,{\text{emission}} = \mathop \sum \limits_{{{\text{i}} = 1}}^{{\text{n}}} \frac{{({\text{F}}_{{\text{i}}} + {\text{F}}_{i + 1} )}}{2} \times ({\text{t}}_{{{\text{i}} + 1}} - {\text{t}}_{{\text{i}}} ) \times 24$$

where F is the N_2_O emission flux (μg N_2_O–N m^−2^ h^−1^), i is the ith measurement, (t_i+1_ − t_i_) is the number of days between two adjacent measurements, and *n* is the total number of the measurements. Annual N_2_O emissions were calculated between the fertilization dates of each successive year.

The SOC stock (Mg ha^−1^) in the topsoil was calculated as:

4 $${\text{C}}_{{{\text{stock}}}} = {\text{SOC}} \times {\text{BD}} \times {\text{D}} \times 10,$$

where BD is soil bulk density (g cm^−3^), D is the depth of the topsoil (0.2 m).

The topsoil SOC sequestration rate (SOCSR) (Mg ha^−1^ yr^−1^) was estimated using the following equation:

5 $${\text{SOCSR}} = \left( {{\text{C}}_{{{\text{stock2015}}}} - {\text{C}}_{{{\text{stock2012}}}} } \right) \times {\text{t}}^{ - 1} ,$$

where C_stock2015_ and C_stock2012_ are the SOC stocks in 2015 and 2012, respectively, and t is the duration of the experiment (years).

Statistical analyses were performed using SPSS 13.0 (SPSS, Chicago, USA). The differences in cumulative N_2_O emissions and maize yields within a year, and other factors among treatments were assessed using one-way Analysis of Variance (ANOVA) with least significant difference post-hoc tests and a 95% confidence limit. The effects of different treatments, years, and their interactions on N_2_O emission, maize yield and aboveground biomass were examined using one-way repeated measures ANOVA. Pearson correlation analysis was used to analyze the relationships between cumulative N_2_O emissions and precipitation (N = 12 (three data each year, four years)), as well as N_2_O flux and soil available nitrogen content.

### Statements of research involving plants

It is stated that the current research on the plants comply with the relevant institutional, national, and international guidelines and legislation. It is also stated that the appropriate permissions have been taken wherever necessary, for collection of plant or seed specimens. It is also stated that the authors comply with the ‘IUCN Policy Statement on Research Involving Species at Risk of Extinction’ and the ‘Convention on the Trade in Endangered Species of Wild Fauna and Flora’.

## Results

### Soil mineral N

Soil NH_4_^+^–N and NO_3_^−^–N concentrations were shown in Fig. S1. The contents of soil NH_4_^+^–N and NO_3_^−^–N increased significantly after fertilization, and gradually decreased after reaching the maximum value. No significant difference in soil NH_4_^+^–N concentrations was found between NPKM and NPKI + M treatments except August 13th 2012. Soil NO_3_^−^–N contents of NPKI + M treatment on June 12th and July 4th 2014 were significantly higher than that of the NPKM treatment.

### Maize grain yield and aboveground biomass

Across the four-year observation period, although the yearly average of maize yield of AM amendment treatment (NPKM and NPKI + M) had an increasing trend relative to NPK treatment, the repeated measurement analysis of variance showed that the difference between these treatments was not significant (*p* > 0.05, Table 1). However, the grain yields were significantly increased in AM amendment treatments (NPKM and NPKI + M) in the fourth year (2015) (Table 1), compared to NPK treatment. During the four-year period, aboveground biomasses of the NPKM and NPKI + M treatments were significantly higher relative to the NPK treatment, by 12.0% and 10.3%, respectively (*p* < 0.05, Table 1). Through repeated-measures ANOVA, the significant interaction was not found between observation years and treatments effect on aboveground biomass.

**Table 1:** Maize grain yields and aboveground biomass from 2012 to 2015 (Mg ha^−1^).

Treatments	Grain yields					Aboveground biomass				
	2012	2013	2014	2015	Mean	2012	2013	2014	2015	Mean
NPK	11.62 ± 0.54 b	11.74 ± 0.88 a	11.60 ± 0.92 b	11.38 ± 0.37 b	11.58 ± 0.44 a	23.22 ± 1.11 a	22.09 ± 1.63 a	20.41 ± 2.07 b	22.44 ± 0.88 b	22.04 ± 0.84 b
NPKM	12.27 ± 0.25 ab	13.13 ± 0.75 a	13.86 ± 0.89 a	12.60 ± 0.36 a	12.96 ± 0.47 a	23.70 ± 1.08 a	24.70 ± 1.32 a	26.11 ± 1.09 a	24.20 ± 0.63 a	24.68 ± 0.48 a
NPKI + M	12.55 ± 0.53 a	12.36 ± 1.53 a	13.32 ± 0.82 ab	12.29 ± 0.44 a	12.63 ± 0.76 a	24.18 ± 2.46 a	23.70 ± 1.97 a	25.11 ± 0.86 a	24.23 ± 0.56 a	24.31 ± 1.25 a

Different lowercase letters indicate significant differences (p < 0.05). “with” the same letters were not significantly different (p > 0.05).

### N_2_O flux and related nitrification and denitrification gene abundance

**Figure 3:**
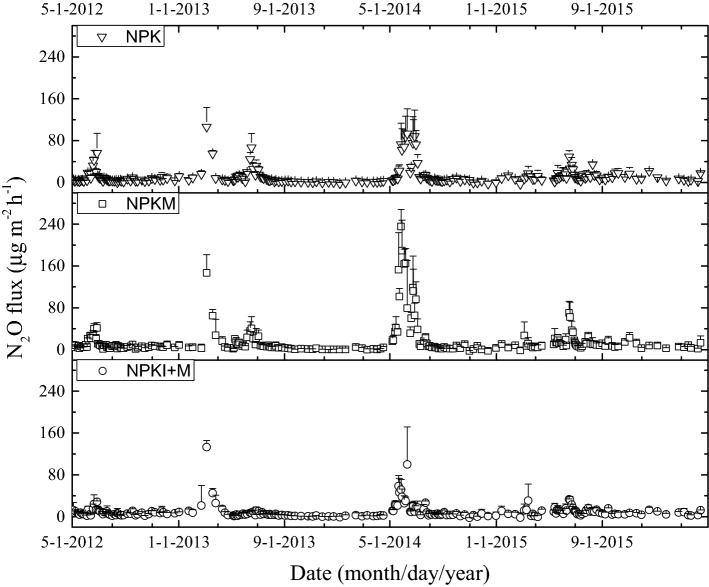
Seasonal variations of N_2_O fluxes in NPK, NPKM and NPKI + M treatments from May 2012 to April 2016. Error bars represent the standard deviation (n = 3).

Seasonal variations in soil N_2_O flux are shown in Fig. 3. The highest N_2_O fluxes typically occurred after fertilizer application and occasionally coincided with freeze–thaw events in 2012/2013 (Fig. 3). The highest N_2_O flux (235.6 μg m^−2^ h^−1^) was observed from the NPKM treatment plot on May 27th 2014, while it was significantly mitigated by the DMPP amendment in NPKI + M treatment (51.3 μg m^−2^ h^−1^).

Relative to the NPK treatment, the cumulative N_2_O emissions from the NPKM plot was significantly increased by 25.8% on average (*p* < 0.05, Table 2). However, in three of four years, N_2_O emissions were not statistically different between the NPK and NPKM treatments. During 2014/2015, NPKM increased N_2_O emissions by 63.0% relative to the NPK treatment (*p* < 0.05). Compared to the NPKM treatment, the addition of DMPP (NPKI + M) significantly decreased annual N_2_O emissions by 51.9%, 54.4%, and 22.5% in 2013/2014, 2014/2015 and 2015/2016, respectively. Across the four-year observation period, the N_2_O emissions decreased by 32.5% in NPKI + M treatment, compared with the NPKM treatment (Table 2).

**Table 2:** Annual cumulative fluxes of N_2_O (kg N ha^−1^) under different treatments through the experimental period (2012–2015).

Treatment	2012–2013	2013–2014	2014–2015	2015–2016	Mean
NPK	1.05 ± 0.17 a	0.40 ± 0.14 a	1.04 ± 0.27 b	0.87 ± 0.12 a	0.84 ± 0.07 b
NPKM	1.26 ± 0.20 a	0.41 ± 0.08 a	1.70 ± 0.40 a	0.86 ± 0.01 a	1.06 ± 0.05 a
NPKI + M	1.22 ± 0.07 a	0.20 ± 0.06 b	0.78 ± 0.19 b	0.66 ± 0.01 b	0.71 ± 0.06 c

Mean ± standard deviation (n = 3). Different lowercase letters in one column indicate significant difference among treatments (*p* < 0.05).

Significant linear negative relationships between precipitation and N_2_O emission in growing season (N = 12, *p* < *0.05*) and significant positive relationships between precipitation and N_2_O emission in non-growing season were found (N = 12, *p* < *0.01*). Correlation analysis showed that N_2_O emission fluxes had a very significant positive correlation with the contents of NH_4_^+^–N and NO_3_^−^–N in soil.

The results of nitrification and denitrification functional gene abundance were shown in Table 3. Compared with NPK, NPKM significantly increased the AOB *amoA* and *nosZ* gene abundance by 88% and 172%, respectively. There was no significant difference in AOB *amoA* and *nosZ* gene abundance between NPK and NPKI + M treatments.

**Table 3:** Ammonia oxidizers and denitrifier functional gene abundance (copies g^−1^ of dry soil).

Treatment	AOA *amoA*	AOB *amoA*	*nirS*	*nirK*	*nosZ*
NPK	9.73E + 06 a	4.87E + 06 b	3.17E + 06 a	5.17E + 05 b	4.55E + 06 b
NPKM	1.46E + 07 a	9.16E + 06 a	3.88E + 06 a	1.20E + 06 ab	1.24E + 07 a
NPKI + M	1.17E + 07 a	5.88E + 06 ab	3.46E + 06 a	1.27E + 06 a	6.37E + 06 b

Values followed by different lowercase letters at the same column indicated significant difference (P < 0.05) among the treatments.

### Soil organic carbon sequestration rate

The SOC content was 9.0 g kg^−1^ at the beginning of the experiment in 2012. Relative to the NPK treatment, SOC content was significantly elevated (by 24.1%) in the NPKI + M treatment after four years of manure application (*p* < 0.05). The annual topsoil SOCSR for the NPKM and NPKI + M treatments was 0.57 Mg ha^−1^ yr^−1^ and 1.02 Mg ha^−1^ yr^−1^, respectively (Table 4). Compared to the NPK treatment, the NPKM and NPKI + M treatments significantly increased SOCSR, respectively (*p* < 0.05, Table 4).

**Table 4:** TN, SOC, C_stock_ and SOCSR at 0–20 cm soil depth after four year’s different fertilization treatments.

Treatment	TN (g kg^−1^)	SOC (g kg^−1^)	C_stock (Mg ha_^−1^_)_	SOCSR^a^ (Mg C ha^−1^ yr^−1^)
NPK	0.93 ± 0.04 b	8.40 ± 0.57 b	20.99 ± 1.42 b	− 0.61 ± 0.38 b
NPKM	1.06 ± 0.09 a	9.64 ± 0.77 ab	24.10 ± 1.92 ab	0.57 ± 0.45 a
NPKI + M	1.01 ± 0.06 ab	10.42 ± 0.69 a	26.05 ± 1.72 a	1.02 ± 0.44 a

^a^The SOCSR was estimated from April 2012 to October 2015.

Values followed by different lowercase letters at the same column indicated significant difference (*P* < 0.05) among the treatments.

## Discussion

### N_2_O emissions

Large inter-annual variations in N_2_O emissions were observed during the study period. Xia et al. and Cayuela et al. reported that N_2_O emission is affected by soil characteristics, climatic conditions, and crop management measures^19,20^. In this study, according to the relationships between precipitation amount and N_2_O emissions, the precipitation amount might be one of the most important controlling factors on N_2_O emissions, especially in the AM addition treatment. Meanwhile, the precipitation distribution might also be an important factor for N_2_O flux. There was a positive correlation between N_2_O flux and soil available N (NH_4_^+^–N and NO_3_^−^–N), indicating that the coupling of water and nitrogen was one of the reasons for the higher N_2_O emissions. Generally speaking, precipitation before and after the fertilization period (plenty available N as shown in Fig. S1) is prone to cause higher N_2_O emissions, such it was in 2014/2015. While in the later growing season (less available N as shown in Fig. S1), even if large precipitation happened, it will not cause higher N_2_O emissions, such it was in August of each year. This may be because the continuous consumption of N in the soil (such as absorption by maize, volatilization, and runoff, etc.) resulted in a decrease in available N in the soil, which ultimately reduced the release of N_2_O. Therefore, the results of our study showed that the distribution and amount of precipitation had a significant effect on N_2_O emissions in a rainfed cropping system, which is consistent with the results reported in previous studies^21^.

In average over 4 years, the addition of AM (NPKM) significantly increased soil N_2_O emissions relative to the control treatment (NPK), which is consistent with previous studies^14,22^. Specifically, N_2_O emissions were 63.0% higher with the addition of AM (NPKM) in 2014/2015 (*p* < 0.05). The higher N_2_O emission recorded for the NPKM treatment might be explained with two key mechanisms: Firstly, the total N input is higher in the NPKM treatment (mean = 346 kg N ha^−1^) than in the NPK treatment (mean = 220 kg N ha^−1^). Previous studies have reported a positive correlation between nitrogen application rates and N_2_O emissions^23,24^, although cumulative N_2_O emission may have an upper threshold under increasing organic nitrogen inputs^14^. Secondly, the long-term organic manure application can increase the total organic C and soil availability of DOC^25,26^, which could stimulate microbial activity and N_2_O production in soil^27^.

In three of the four observation years, cumulative N_2_O emissions did not differ between the NPK and NPKM treatments despite the much greater N application in the NPKM plot, and this phenomenon is consistent with previous studies^12,28^. Organic fertilizer provides organic C substrate for microbial growth, so it promotes microbial N assimilation. This effect usually leads to a strong competition for NH_4_^+^ between heterotrophic microorganisms and autotrophic nitrifiers, mitigating the yield of N_2_O^29^. However, the input of organic C and N may promote the growth of active microorganisms and consume O_2_ in soil pores, resulting in the formation of micro-anaerobic environments, stimulate denitrification and produce N_2_O^7,30^. In this study, the NPKM treatment increased the occurrence of the *nosZ* gene by 172% (supplementary materials, Table 3), relative to the NPK treatment, indicating a higher portion of N_2_O had been reduced to N_2_ in NPKM treatment. Meanwhile, the higher AOB amoA gene was also found in NPKM treatment, which might induce much N_2_O formation. Therefore, considering the combined effects of the above nitrification and denitrification, there was no significant difference in N_2_O emissions between NPK and NPKM in 2015 in our study. Overall, our results suggest that, in the rainfed maize cropping system, the combined application of inorganic fertilizer and AM might promote the emission of N_2_O in comparison to inorganic fertilizer applied alone.

The amounts of inorganic N (220 kg N ha^−1^) and AM (15 Mg ha^−1^) were selected in this study according to the usual amounts of fertilizers applied by local farmers. The addition of AM brings in a large amount of organic N (mean = 126 kg N ha^−1^) in NPKM treatment, and the total N applied in NPKM treatment was much higher (by 57.3%) than that in NPK treatment. In addition to increasing N_2_O emissions and maize biomass, a large part of the applied N was stored in the soil according to TN data (Table 4). Further studies should be conducted to investigate the long-term application of AM on N loss in a maize-soil system.

The addition of DMPP (NPKI + M) significantly decreased cumulative N_2_O emissions relative to the NPKM treatment, which is consistent with previous studies^9,11,31^. The observed percentage in N_2_O emissions reduction ranged between 22.5% and 54.4%, which is comparable to other studies applying DMPP including a reduction of 24% reported by Huérfano et al.^32^ and 53% reported by Weiske et al.^9^. Based on a review of the literature on NI application, Akiyama et al.^33^ reported that the application of NI reduces N_2_O emissions by an average of 38%. Furthermore, Qiao et al.^34^ reported that NI application could increase NH_3_ emission by 20%. Indirect N_2_O losses (i.e., NO_3_^−^–N leaching and NH_3_ volatilization) may sometimes be greater than direct N_2_O emission^35,36^. The application of organic fertilizer usually has significant effect on soil NH_3_ emission^36^, but the effect of NI, AM and NPK combined application on NH_3_ emission has not been well elucidated. Therefore, it is necessary to evaluate the effect of NI application combined with organic fertilizer on nitrogen loss as a whole in further studies.

In this study, the results showed that NPKI + M treatment could significantly reduce N_2_O emissions compared to NPKM treatment. However, due to lack of the NPK + NI treatment, the contribution of combined application of nitrification inhibitor (DMPP) and inorganic fertilizers to the reduction of soil N_2_O emissions was not measured and evaluated. Therefore, in order to elucidate the process, in addition to add the NPK + NI treatment, the stable isotope labeling technique was suggested to be used to clarify the source and proportion of reduced N_2_O in future studies^37,38^.

### Maize yield and SOCSR

Addition of AM significantly increased (10.7% and 8.0% for NPKM and NPKI + M, respectively) the maize yields in the fourth year, which is comparable to the study of Li et al.^39^ conducted in Northeast China. On one hand, there was more N provided in AM amendment treatment in comparison to NPK treatment. On the other hand, the organic form of N was released later in the growing season of maize (especially in 2014 and 2015, Fig. S1), which provided a better match between N supply and maize requirement in comparison to NPK treatment. In comparison, maize yields were not significantly affected by DMPP application, as has also been reported^31^.

The results showed that long-term application of inorganic fertilizers induced the loss of SOC, since C inputs obtained only from maize residue were smaller than C loss in inorganic fertilizer treatment. It has also been reported in other studies in Northeast China, in which a declined SOC was found in inorganic fertilizer treatment^39,40^. Therefore, in our opinion, for the sustainable development of agriculture in Northeast China, it is necessary to apply AM with inorganic fertilizers. The annual SOCSR in this study was similar to a multi-site study of manure application in a mono-cropping system reported by Zhang et al.^41^ and a soybean and maize rotation system in Northeast China by Ding et al.^42^. The results suggest that the sequestration of SOC might be mainly associated with the direct C supply from AM and the indirect C supply through higher maize yields^43^. Application of organic manure is an effective agricultural practice for enhancing SOC storage in the maize cropping system^44,45^. It is necessary to further study the processes and mechanisms of SOC sequestration induced by DMPP application.

Based on the results of maize grain yield and aboveground biomass, NPKM would be used to achieve higher maize yield and aboveground biomass, but it would increase N_2_O emission of maize production. Compared with NPK, NPKM did not significantly increased the content of SOC, while SOC were significantly increased by combined inorganic and organic fertilizer application with DMPP. The results of this study suggest that increasing SOC and maize yield, as well as N_2_O mitigation can be simultaneously achieved by the combined application of inorganic and organic fertilizer with DMPP. It is necessary to measure the changes of SOC and N_2_O emissions at the same time when formulating the optimal management measures for sustainable maize production.

## Conclusions

Long term application of inorganic fertilizers led to the loss of SOC. Generally speaking, applying animal manure is considered to be an effective way to improve soil SOC. However, there is a risk of enhanced N_2_O emission with manure application. Through a consecutive four-year field experiment on Luvisol soil in Northeast China, our results showed that the combined application of NI, such as DMPP, inorganic fertilizer and animal manure into soil should be recommended in Northeast China, as it could not only mitigate N_2_O emissions but also increase maize yield and SOC sequestration rate.

## Supplementary Information

Supplementary Information.
