# Supplementary material for: Effects of animal manure and nitrification inhibitor on N2O emissions and soil carbon stocks of a maize cropping system in Northeast China
Source: Sci Rep. 2022 Sep 8;12:15202. doi: 10.1038/s41598-022-19592-9 (PMC9458636; doi:10.1038/s41598-022-19592-9)
Supplement: Supplementary file 1 — Supplementary Information. [file 41598_2022_19592_MOESM1_ESM.docx]

Supplementary materials

Effects of animal manure and nitrification inhibitor on N_2_O emissions and soil carbon stocks of a maize cropping system in Northeast China

Dan Dong^a,b,c,1^, Weichao Yang^a,1^, Hao Sun^a^, Shuang Kong^a^, Hui Xu^a,*^

*^a^Key Laboratory of Pollution Ecology and Environmental Engineering, Institute of Applied Ecology, Chinese Academy of Sciences, Shenyang 110016, China*

*^b^Jiangsu Collaborative Innovation Center of Regional Modern Agriculture and Environmental Protection/Jiangsu Key Laboratory for Eco-Agriculture Biotechnology Around Hongze Lake, Huaiyin Normal University, Huai’an 223300, China*

*^c^University of Chinese Academy of Sciences, Beijing 100049, China*

*^*^Corresponding author.* E-mail address: *xuhui@iae.ac.cn*

*^1^These authors contributed equally to this work and should be considered co-ﬁrst authors.*

**1. Characteristics of pig manure during the four years study**

After air drying, pig manure was ground to pass through a 0.15 mm sieve to measure total C and N contents with elemental analyzer (Vario EL Ⅲ, Elementar, Germany).

Table S1 Characteristics of pig manure during the four years study

| Years | C (%) | N (%) | C/N ratio |
| --- | --- | --- | --- |
| 2012 | 19.88 | 1.56 | 12.74 |
| 2013 | 23.87 | 2.15 | 11.11 |
| 2014 | 21.08 | 2.50 | 8.43 |
| 2015 | 20.62 | 2.76 | 7.48 |

**2. Seasonal variations of soil available NH_4_^+^-N and NO_3_^-^-N during experimental period**

Fig. S1 Seasonal variations of soil available NH_4_^+^-N and NO_3_^-^-N in NPK, NPKM and NPKI + M treatments from May 2012 to April 2016. Error bars represent the standard deviation (n = 3).
